# Proteomic Investigation of the Antibacterial Mechanism of Cefiderocol against Escherichia coli

**DOI:** 10.1128/spectrum.01093-22

**Published:** 2022-08-18

**Authors:** Gao-Fei Du, Yu Dong, Xiaolu Fan, Ankang Yin, Yao-Jin Le, Xiao-Yan Yang

**Affiliations:** a Key Laboratory of Laboratory Diagnostics, Medical Technology School, Xuzhou Medical University, Xuzhou, Jiangsu, China; b Department of Bioengineering, Zhuhai Campus of Zunyi Medical University, Zhuhai, Guangdong, China; c Fujian Agriculture and Forestry University, Fuzhou, China; d NHC Key Laboratory of Technical Evaluation of Fertility Regulation for Non-human Primate (Fujian Maternity and Child Health Hospital), Fuzhou, China; University of Exeter

**Keywords:** cefiderocol, *E. coli*, ROS, NADH, iron ions

## Abstract

This study aimed to investigate the antibacterial mechanism of cefiderocol (CFDC) using data-independent acquisition quantitative proteomics combined with cellular and molecular biological assays. Numerous differentially expressed proteins related to the production of NADH, reduced cofactor flavin adenine dinucleotide (FADH_2_), NADPH and reactive oxygen species (ROS), iron-sulfur cluster binding, and iron ion homeostasis were found to be upregulated by CFDC. Furthermore, parallel reaction monitoring analysis validated these results. Meanwhile, we confirmed that the levels of NADH, ROS, H_2_O_2_, and iron ions were induced by CFDC, and the sensitivity of Escherichia coli to CFDC was inhibited by the antioxidant vitamin C, *N*-acetyl-l-cysteine, and deferoxamine. Moreover, deferoxamine also suppressed the H_2_O_2_ stress induced by CFDC. In addition, knockout of the NADH-quinone oxidoreductase genes (*nuoA*, *nuoC*, *nuoE*, *nuoF*, *nuoG*, *nuoJ*, *nuoL*, *nuoM*) in the respiratory chain attenuated the sensitivity of E. coli to CFDC far beyond the effects of cefepime and ceftazidime; in particular, the E. coli BW25113 Δ*nuoJ* strain produced 60-fold increases in MIC to CFDC compared to that of the wild-type E. coli BW25113 strain. The present study revealed that CFDC exerts its antibacterial effects by inducing ROS stress by elevating the levels of NADH and iron ions in E. coli.

**IMPORTANCE** CFDC was the first FDA-approved siderophore cephalosporin antibiotic in 2019 and is known for its Trojan horse tactics and broad antimicrobial activity against Gram-negative bacteria. However, its antibacterial mechanism is not fully understood, and whether it has an impact on *in vivo* iron ion homeostasis remains unknown. To comprehensively reveal the antibacterial mechanisms of CFDC, data-independent acquisition quantitative proteomics combined with cellular and molecular biological assays were performed in this study. The findings will further facilitate our understanding of the antibacterial mechanism of CFDC and may provide a theoretical foundation for controlling CFDC resistance in the future.

## INTRODUCTION

Rising concerns of bacterial infections have become a major threat to global human health, especially multidrug-resistant (MDR) Gram-negative pathogens, which have challenged the clinical efficacy of many antibiotics (1–4). The WHO has designated carbapenem-resistant (CR) strains of *Enterobacterales*, Pseudomonas aeruginosa, and Acinetobacter baumannii and third-generation cephalosporin-resistant *Enterobacterales* as “priority 1: critical” pathogens, which emphasizes the need for comparatively more potent antibiotics (5). However, since the strategy for developing novel anti-MDR drugs with antibacterial mechanisms different from those of current antibiotics is laborious and time-consuming, modifying the structure of existing antibiotics offers better feasibility in the development of novel antibacterial agents.

Siderophores, including enterobactin (catecholate), desferrioxamine (hydroxamate), and pyoverdine (mixed type), are small organic iron-chelating molecules secreted by bacteria (6–8). All siderophores enter target bacteria via specific iron transport systems (9–12). Siderophore-antibiotic conjugate design is a new drug design strategy that links an antibiotic to a microbial siderophore or siderophore mimic. This “Trojan horse” design can not only help antibiotics overcome the resistant bacterial cell wall permeability barrier but also increase accumulation of antibiotics in the periplasmic space, which is the location of their targets (13–21).

Cefiderocol (CFDC) is the first FDA-approved parenteral siderophore cephalosporin antibiotic with a broad range of antimicrobial activity against Gram-negative bacteria, including CR *Enterobacterales* and nonfermenters (such as Pseudomonas aeruginosa, Acinetobacter baumannii, *Stenotrophomonas*, and *Burkholderia*) (22, 23). The basic structural features of CFDC are designed from ceftazidime (CAZ) and cefepime (CPM), enabling CFDC to inhibit peptidoglycan cell wall biosynthesis and withstand hydrolysis by β-lactamases. The unique chemical component is the addition of a catechol moiety on the C-3 side chain, which chelates ferric (Fe-III) iron to mimic natural siderophores (24). This process could increase the concentration of CFDC in the periplasmic space and enhance the activity of CFDC relative to other cephalosporins. However, the antibacterial mechanism of CFDC is not completely understood, especially whether iron homeostasis in cells is disrupted by CFDC.

Quantitative proteomics provides a systemic tool to investigate the antibacterial mechanisms of antibiotic and natural products (25–27). In this study, data-independent acquisition (DIA) quantitative proteomics combined with bioinformatics was used to investigate protein expression changes and provide a more comprehensive systemic understanding of the potential antibacterial mechanisms of CFDC against E. coli.

## RESULTS

### Overview analysis of the E. coli proteome after CFDC treatment.

**FIG 1:**
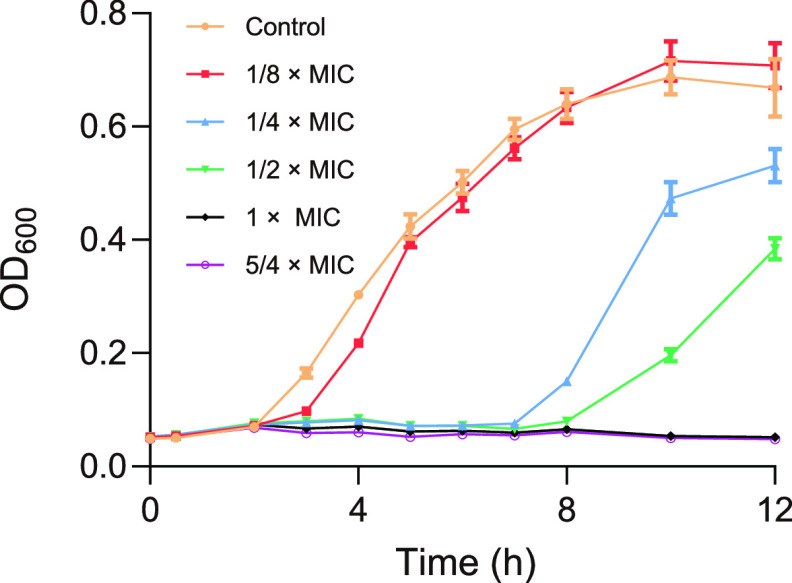
Growth curves of E. coli with different concentrations of CFDC or without CFDC (control) treatment. The 1/8× MIC, 1/4× MIC, 1/2× MIC, 1× MIC, and 5/4× MIC values of CFDC against E. coli were 0.1, 0.2, 0.4, 0.8, and 1.0 μg/mL, respectively. The data represent the means of three cultures; error bars indicate SD.

**FIG 2:**
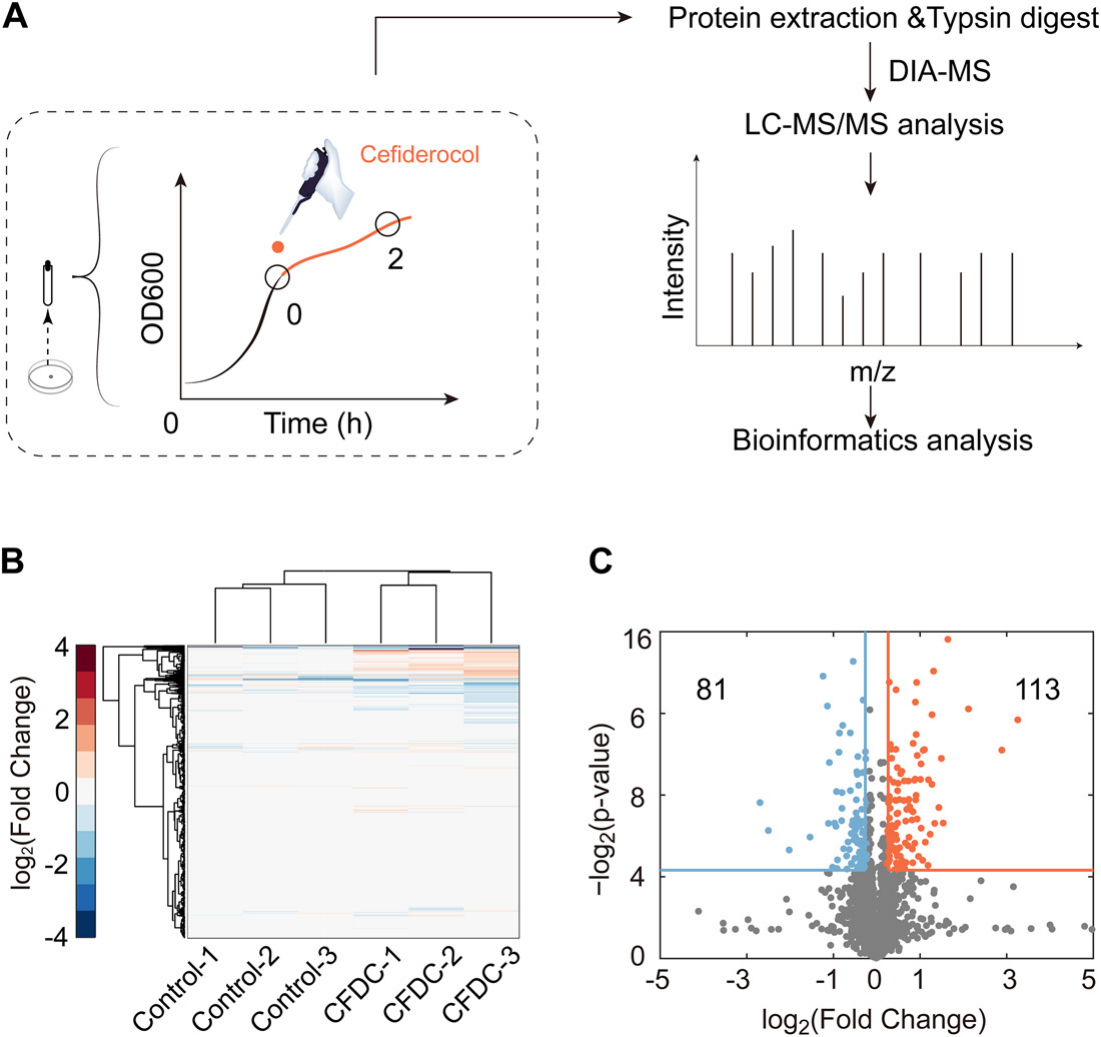
Workflow of the proteomic experiment and statistical analysis of proteomic changes in E. coli treated with CFDC. (A) Workflow of DIA MS analysis. (B) Hierarchical clustering analyses of all six samples. (C) Volcano plots of CFDC-treated groups.

To assess the antibacterial activity of CFDC against E. coli, we monitored the MIC and the growth curves of E. coli treated with different concentrations of CFDC. The MIC of CFDC against E. coli was 0.8 μg/mL, and the growth curves of E. coli treated with 0, 1/8× MIC, 1/4× MIC, 1/2× MIC, 1× MIC, and 5/4× MIC CFDC is shown in Fig. 1. In order to minimize bacterial death caused by CFDC and observe real changes in the abundance of proteins, 1/2 × MIC (0.4 μg/mL CFDC) was chosen to treat E. coli in the logarithmic phase for the proteomics assay in this study. DIA-based quantitative proteomics was applied to analyze the global protein alteration of E. coli from the 0 h (control) and 2 h CFDC-treated groups (Fig. 2A). Herein, a total of 2,498 proteins were identified from six samples that were three replicates of two different conditions. Hierarchical clustering analyses showed that all six samples were clearly clustered into two classes, the control and CFDC-treated groups, indicating the reliability of our proteome data (Fig. 2B). Notably, proteins with expression |fold change| ≥1.2 and a *P* value of <0.05 in treated groups were considered significant as differentially expressed proteins (DEPs). As shown in the volcano plots, 194 DEPs, including 114 upregulated proteins and 81 downregulated proteins, were identified in the samples with CFDC-2 h treatment (Fig. 2C; see also Table S1 in the supplemental material).

### Bioinformatics analysis.

**FIG 3:**
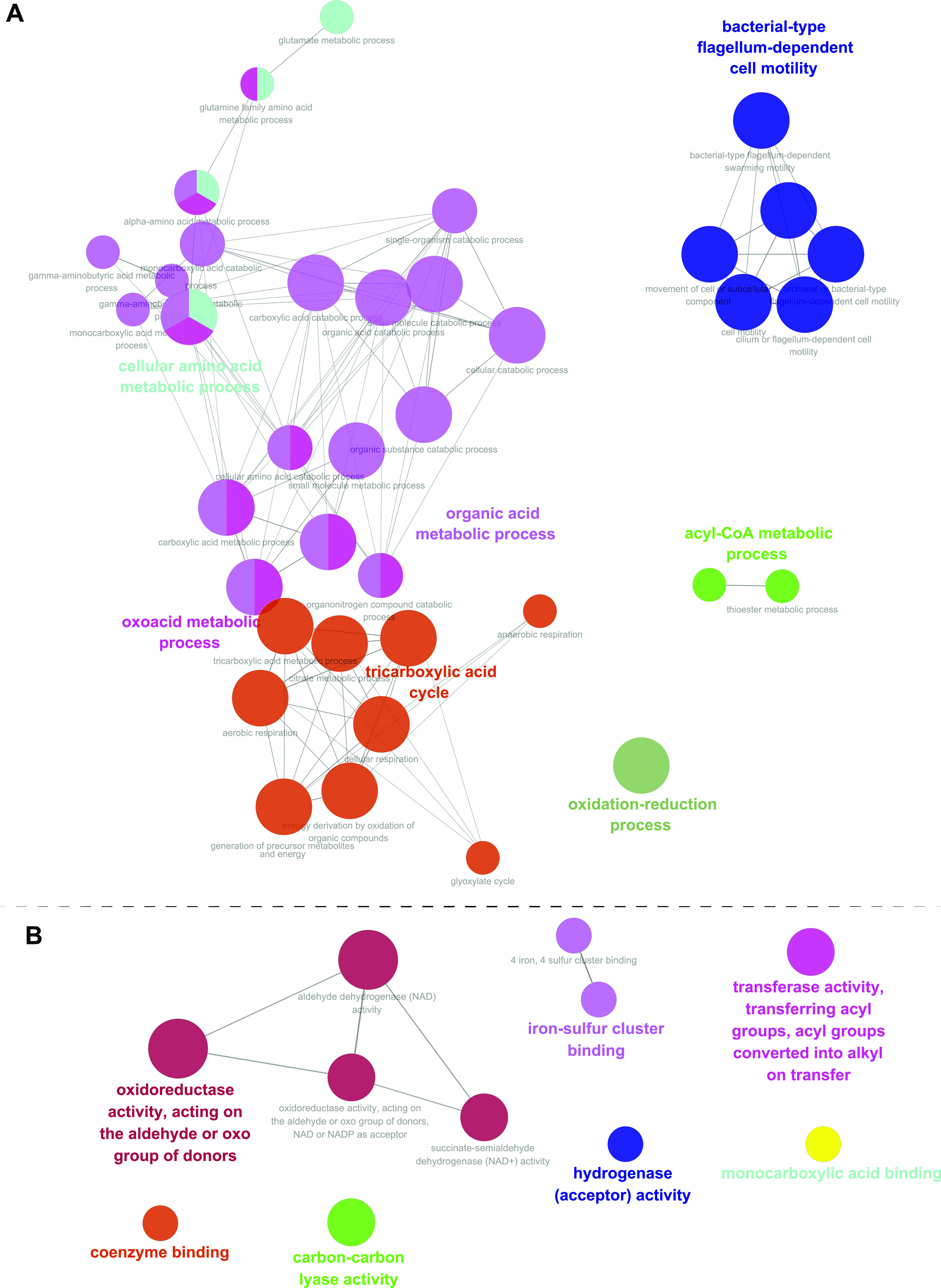
GO analysis of DEPs. (A) GO biological processes of DEPs. (B) GO molecular functions of DEPs. The sizes of the nodes indicate the *P* value involved in the GO term, the connecting lines between each node indicate the correlation between each node (GO terms), and the different colors of nodes represent different GO groups.

To explore the pathways and molecular functions involved in the antibacterial effects of CFDC in E. coli, we performed Gene Ontology (GO) and KEGG analysis for all DEPs. As shown in Fig. 3A, these DEPs were mainly involved in cellular amino acid, organic acid, and oxoacid metabolic processes; tricarboxylic acid cycle (TCA cycle); acyl coenzyme A (acyl-CoA) metabolic processes; oxidation-reduction processes; and cell motility biological processes. GO molecular function enrichment analysis indicated that DEPs are mainly involved in oxidoreductase activity (acting on the aldehyde or oxo groups of donors, with NAD or NADP as the acceptor), iron binding (hydrogenase [acceptor] activity, iron-sulfur cluster binding), penicillin binding (monocarboxylic acid binding), transferase activity (transferring acyl groups, acyl groups converted into alkyl on transfer), and carbon-carbon lyase activity (Fig. 3B).

**FIG 4:**
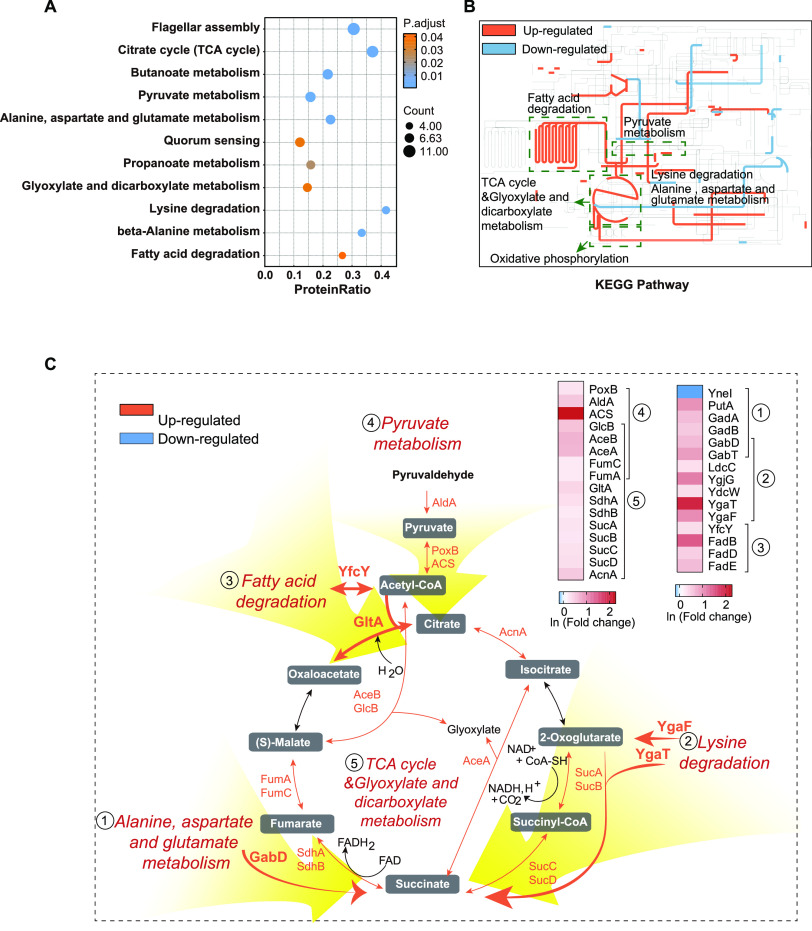
KEGG pathway analysis of DEPs. (A) KEGG pathway enrichment analysis of DEPs in CFDC-treated groups. (B) Metabolic pathways in CFDC-treated groups. (C) Schematic diagram of DEPs involved in the main metabolic pathways in the CFDC-treated groups.

Additionally, KEGG enrichment analysis showed that the DEPs were mainly involved in carbohydrate metabolism (TCA cycle, glyoxylate and dicarboxylate metabolism, pyruvate metabolism, butanoate metabolism, and propanoate metabolism), fatty acid degradation, and amino acid metabolism (alanine, aspartate, and glutamate metabolism; lysine degradation; and β-alanine metabolism) (Fig. 4A and B). Notably, most of these metabolic pathways were upregulated and showed an obvious trend to enhance the TCA cycle and NADH and reduced cofactor flavin adenine dinucleotide (FADH_2_) biosynthesis (Fig. 4C). Conversely, all DEPs involved in flagellar assembly (FlgB, FlgC, FlgD, FlgE, FlgF, FlgG, FlgI, FlgL, FlhD, FliC, FliS) were downregulated; moreover, semisolid agarose assays also validated that bacterial motility was decreased upon CFDC treatment (see Fig. S1A in the supplemental material).

Taken together, the bioinformatics analysis indicated that CFDC exerts its antibacterial effect by affecting energy synthesis; oxidation-reduction processes; NADH, FADH_2_, and NADPH biosynthesis; iron binding; penicillin binding; and cell motility in E. coli.

### NADH and ROS generation induced by CFDC.

**FIG 5:**
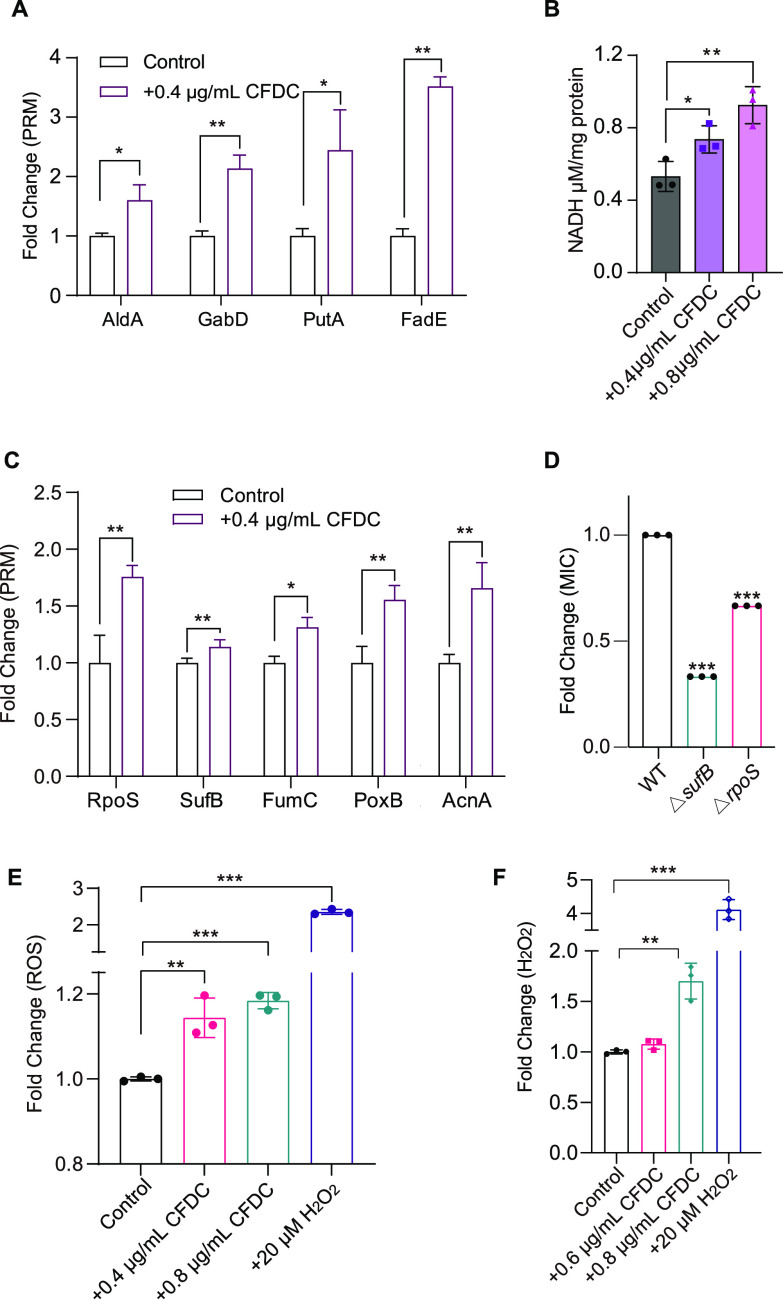
NADH and ROS generation induced by CFDC. (A) DEPs involved in catalyzing the synthesis of NADH (AldA and PutA), NADPH (GabD), and FADH (FadE) were validated by using PRM. (B) Levels of NADH with or without CFDC treatment. (C) DEPs involved in resistance to ROS stress were validated by using PRM. (D) Fold change of MIC of CFDC in wild type and Δ*sufB* and Δ*rpoS* gene knockout strains involved in response to ROS stress. Fold change in the levels of ROS (E) and H_2_O_2_ (F) in cells with or without CFDC treatment. Data in panels A, C, and D were analyzed by using unpaired Student's *t* test, and data in panels B, E, and F were analyzed by using the one-way ANOVA test with Dunnett's correction; error bars indicate SD values. *, *P *< 0.05; **, *P *< 0.01; ***, *P *< 0.001.

As shown in Fig. 4C and Table S2 in the supplemental material, many DEPs involved in catalytic synthesis of NADH (SucA, SucB, AldA, YdcW, and PutA), FADH (SdhA, SdhB, and FadE), and NADPH (GabD) and conversion of NADPH to NADH (SthA) were upregulated. The parallel reaction monitoring (PRM) as a target proteomics method was widely used for validation of DEPs from nontarget proteomics, e.g., data-independent acquisition mass spectrometry (DIA-MS). To validate the reliability of the DIA-MS results, several proteins were selected based on the functional analysis to perform a PRM experiment. Among these DEPs, AldA, GabD, PutA, and FadE were validated by using PRM (Fig. 5A). Even more significant is the fact that the levels of NADH were increased in the 1/2× MIC CFDC-treated groups compared to the control group (Fig. 5B). Several studies have reported that NADH, FADH_2_, and NADPH can serve as electron donors and elevate NADH levels to increase reactive oxygen species (ROS) production in cells (28–32). GO enrichment analysis also revealed that the oxidation-reduction process was changed in the CFDC-treated groups. Meanwhile, many DEPs involved in resistance to ROS stress, including RNA polymerase sigma factor (RpoS), FeS cluster assembly protein (SufB), fumarate hydratase class II (FumC), pyruvate dehydrogenase (PoxB), and aconitate hydratase A (AcnA), were also induced by CFDC (33–36), and these results were also validated by PRM (Fig. 5C). Additionally, the MICs of the Δ*sufB* and Δ*rpoS* strains for CFDC were significantly decreased compared to those of the wild-type E. coli strain (Fig. 5D). Importantly, we detected that the levels of ROS and H_2_O_2_ were significantly upregulated in the CFDC-treated groups (Fig. 5E and F). Collectively, these results demonstrated that the NADH and ROS induced by CFDC contributed to the cell death of E. coli.

### Iron overload.

**FIG 6:**
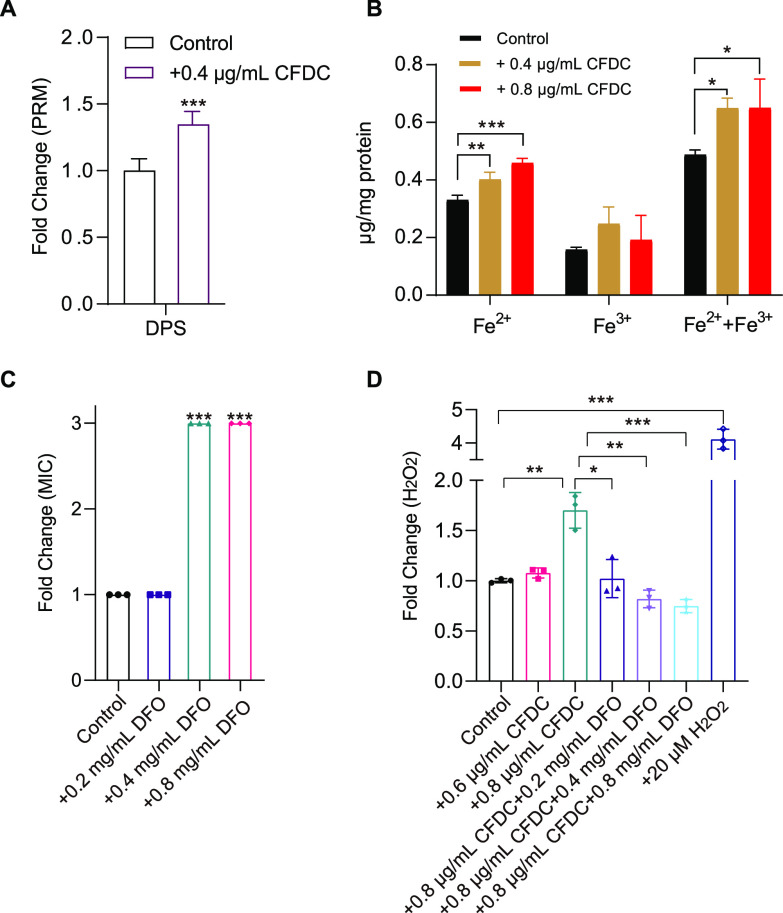
Iron overload. (A) DEPs involved in iron stores (DPS) were validated by PRM. (B) Levels of Fe^2+^, Fe^3+^, and total iron ions (Fe^2+^ and Fe^3+^) in cells with or without CFDC treatment. (C) Fold change in the MIC of CFDC with or without DFO. (D) Fold change of levels of H_2_O_2_ (F) in cells with or without CFDC and combined CFDC and DFO treatment. Data in panel A were analyzed by using unpaired Student's *t* test, and data in panels B to D were analyzed by using the one-way ANOVA test with Dunnett's correction; error bars indicate SD values. *, *P *< 0.05; **, *P *< 0.01; ***, *P* < 0.001.

Since it functions to transport cephalosporins via the iron transport channel, CFDC is significantly different from other cephalosporins. In this study, GO enrichment analysis also showed that iron binding was altered in CFDC-treated groups, which indicated that iron homeostasis might be affected. Meanwhile, the DNA protection during starvation protein (DPS), which has been reported to protect cells from iron overload toxicity, was significantly upregulated and validated by PRM after CFDC treatment (37, 38) (Fig. 6A). Therefore, we detected the iron level in E. coli. As expected, the levels of total iron (the sum of Fe^3+^ and Fe^2+^) and Fe^2+^ were significantly induced by CFDC (Fig. 6B) but not induced by CPM or CAZ (Fig. S1B). To further explore the biological significance of the iron contribution to cell death, we exploited deferoxamine (DFO), which can chelate iron ions, in the experiment. We found that the MIC of CFDC against E. coli was significantly increased 3-fold after DFO was added to CFDC-treated groups compared to that in the control group (Fig. 6C and D). The results indicated that iron overload promoted bacterial killing. Furthermore, we also detected that DFO reduced the levels of H_2_O_2_ in cells induced by CFDC (Fig. 6D). Based on the above results, it seems reasonable to conclude that CFDC can induce iron overload to enhance ROS stress in E. coli.

### CFDC confers stronger ROS stress than CPM and CAZ.

As mentioned above, CFDC induced ROS stress in E. coli, and the molecular structure of CFDC is similar to that of CPM and CAZ except for its unique catechol moiety. These unique structures might mediate the production of ROS stress by iron overload in CFDC-treated groups.

**FIG 7:**
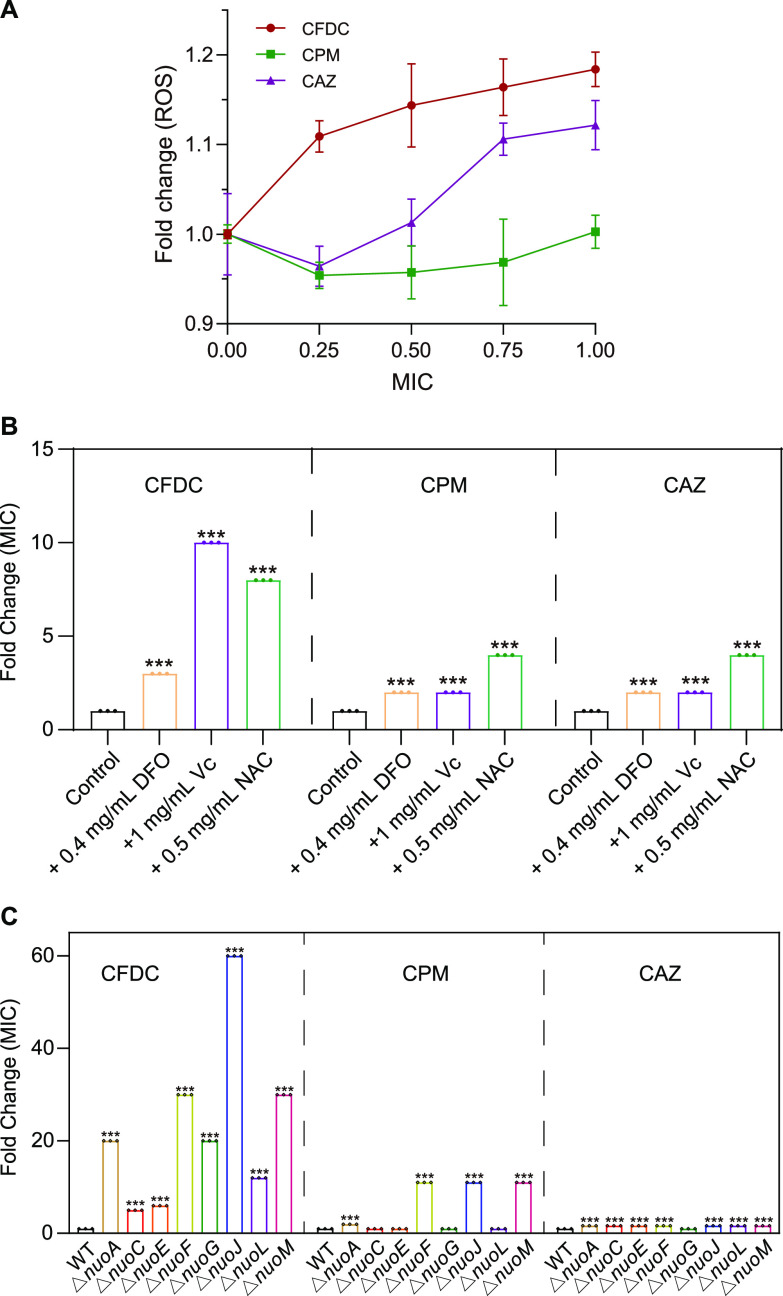
CFDC confers stronger ROS stress than CPM and CAZ. (A) Levels of ROS in the cells after CFDC, CPM, and CAZ treatments. (B) Fold change of MIC of CFDC, CPM, and CAZ with or without DFO, Vc, and NAC. Fold change of MIC of CFDC, CPM, and CAZ with respect to wild type and NADH-quinone oxidoreductase gene knockout strains (Δ*nuoA*, ΔnuoC, Δ*nuoE*, Δ*nuoF*, Δ*nuoG*, Δ*nuoJ*, Δ*nuoL*, and Δ*nuoM* strains), respectively. Data in panels B and C were analyzed by using the one-way ANOVA test with Dunnett's correction; error bars indicate SD values. ***, *P *< 0.001.

Thus, to confirm whether CFDC confers stronger ROS stress than these two structural homologs, we detected levels of ROS in cells after CFDC, CPM, and CAZ treatment, respectively. The result suggested that CFDC induced stronger ROS stress than these two structural homologs at the same relative MIC stress (Fig. 7A). E. coli cells were pretreated with antioxidant vitamin C (V_C_) (1 mg/mL) or *N*-acetyl-l-cysteine (NAC) (0.5 mg/mL) for 1 h prior to CFDC, CPM, or CAZ treatment, and the MIC was determined. Moreover, the MIC of CFDC against E. coli was significantly increased 3-fold, 10-fold, and 8-fold after pretreatment with DFO, V_C_, and NAC, respectively. However, the MIC values of CPM and CAZ increased by only 2-fold, 2-fold, and 4-fold after pretreatment with DFO, V_C_, and NAC, respectively (Fig. 7B).

In addition, NADH-quinone oxidoreductase (respiratory complex I) is considered one of the main sources for ROS in E. coli (39), the *nuo* deletion mutants showed lower ROS production (40). To evaluate the relationship between ROS and these three drugs, we screened a series of mutants of NADH-quinone oxidoreductase genes (*nuoA*, *nuoC*, *nuoE*, *nuoF*, *nuoG*, *nuoJ*, *nuoL*, and *nuoM*) that inhibited ROS stress by interfering with electron transport in the respiratory chain. All NADH-quinone oxidoreductase gene-knockout E. coli strains showed less sensitivity to CFDC than the other two cephalosporins. In particular, the E. coli BW25113 Δ*nuoJ* strain produced 60-fold increases in the MIC of CFDC compared to that of the wild-type E. coli BW25113 strain (Fig. 7C). Taken together, CFDC conferred stronger ROS stress than CPM and CAZ, and the gene mutation in NADH-quinone oxidoreductases led to high levels of CFDC resistance in E. coli.

## DISCUSSION

In recent years, multidrug-resistant infections caused by Gram-negative bacteria have become one of the core reasons for clinical anti-infective treatment failure. CFDC, the first clinically approved siderophore-antibiotic conjugate, has been a research hot spot due to its broad range of antibacterial activity against Gram-negative bacteria and the “Trojan horse strategy,” which was designed to bypass the Gram-negative bacterial cell wall. However, the antibacterial mechanism of CFDC is not well understood. Hence, the *in vitro* study of the mechanism of action of CFDC against E. coli was carried out by using a high-quality DIA-based quantitative proteomics approach.

In this study, we successfully identified 194 DEPs in E. coli treated with CFDC. Bioinformatic analysis showed that energy synthesis; oxidation-reduction processes; NADH, FADH_2_, and NADPH biosynthesis; iron binding; penicillin binding; and cell motility were mainly induced by CFDC (Fig. 3 and 4). The elevated penicillin-binding proteins AmpH, PBP4, and PBP3 indicated the action of cephalosporin in CFDC and validated the reliability of the proteomic data.

Iron, as a key cofactor of enzymes, plays important roles in bacterial proliferation and infection. Our data showed that total iron (the sum of Fe^3+^ and Fe^2+^) and Fe^2+^ were overloaded in the CFDC-treated groups. The elevated Fe^2+^ could promote ROS production by Fenton reaction (41). Moreover, we detected that the ferroptosis inhibitor DFO reduced the sensitivity of CFDC to E. coli (Fig. 6C). In this regard, we speculated that Fe^3+^ might also participate in CFDC-induced bactericidal effect. As described above, the siderophore catechol moiety on the C-3 side chain of CFDC chelated Fe^3+^ into cells, and Fe^3+^ was rapidly reduced to Fe^2+^ by ferric reductase; thus, Fe^3+^ overload promoted increased levels of Fe^2+^, and the Dps protein of E. coli could store elevated Fe^2+^ with H_2_O_2_ as the oxidant (42, 43). Furthermore, the latest study reported that Fe^3+^ could elevate ROS production by promoting the pyruvate cycle (44). In this study, we also identified the same results (Fig. 4C). The DEPs PoxB, ACS, SucA, SucB, SdhA, SdhB, FumA, and FumC in the pyruvate cycle and the levels of NADH and ROS were also increased after exposure to CFDC. Moreover, our proteomic data presented broader changes involved in carbohydrate metabolism, fatty acid degradation, and amino acid metabolism, and these changes suggested an obvious trend to enhance the TCA cycle and NADH and FADH_2_ biosynthesis processes when E. coli was treated with CFDC.

It has been reported that ROS serve important roles in the processes of antimicrobial lethality, in which antibiotic-induced TCA cycle- and respiratory chain-dependent ROS production are involved (45–48). Moreover, ROS stress induced by sublethal concentrations of antibiotics can lead to multidrug resistance (49, 50). Notably, Ye et al. reported that CAZ-induced ROS production was required for CAZ-mediated killing in ceftazidime-resistant Edwardsiella tarda (LTB4-R_CAZ_), and a reduction in ROS production contributed to E. tarda resistance to CAZ (44). In our work, we demonstrated that CFDC confers stronger ROS stress than CPM and CAZ, and knockout of NADH-quinone oxidoreductase genes (*nuoA*, *nuoC*, *nuoE*, *nuoF*, *nuoG*, *nuoJ*, *nuoL*, *nuoM*) in the respiratory chain promoted insensitivity of E. coli to CFDC far beyond the effects of CPM and CAZ. In particular, the E. coli BW25113 Δ*nuoJ* strain produced 60-fold increases in MIC to CFDC compared to the wild-type E. coli BW25113 strain (Fig. 7). These results imply that the alteration of redox imbalance induced by CFDC plays an important role in the antibacterial mechanism of CFDC but also in the emergence of CFDC resistance.

**FIG 8:**
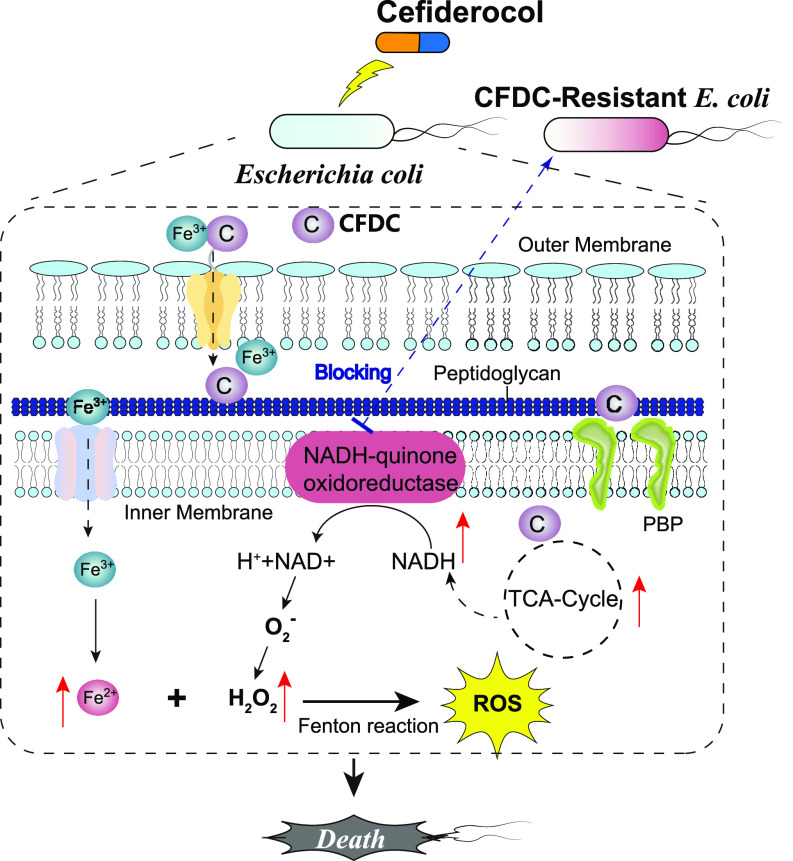
Schematic representation of antibacterial mechanism of CFDC against Escherichia coli.

In conclusion, this study revealed a global proteomic alteration of E. coli after CFDC treatment. Based on the bioinformatic analysis, PRM, and cellular and molecular biological validation, these results suggest that CFDC exerts its antibacterial effects by inducing ROS stress by elevating NADH and iron overload. CFDC conferred stronger ROS stress than the other two cephalosporins, CPM and CAZ, and the gene mutation in NADH-quinone oxidoreductases led to high levels of CFDC resistance in E. coli (Fig. 8). These findings provide knowledge of the antibacterial mechanism of siderophore-antibiotic conjugates and enable new strategies for inhibiting infections by CFDC-resistant human pathogens in the future.

## MATERIALS AND METHODS

### CFDC, bacteria, and culture conditions.

CFDC (S-649266; purity greater than 99.85%) was purchased from MedChemExpress and dissolved in sterile deionized water as a stock solution at 2 mg/mL. Wild-type E. coli strain BW25113 and E. coli knockout strains were obtained from the National BioResource Project (NBRP) (National Institute of Genetics [NIG], Japan). The strains were grown overnight in Luria-Bertani (LB) medium at 37°C and then diluted 1:100 into fresh medium for reactivation.

### MIC determination.

MIC for CFDC was determined by using a microdilution assay in a sterile 48-well plate. The bacteria were diluted to an optical density at 600 nm (OD_600_) of ~0.05, and 500 μL of bacterial solution with drug at different final concentrations was added to each well. After incubating for 24 h at 37°C, the lowest concentration with no visible growth (OD_600_, <0.1) was identified as the MIC (51).

### Protein extraction, digestion, and fractionation.

Activated strains were inoculated into fresh LB medium until an OD_600_ of ~0.6 was observed, and then cell pellets were harvested via centrifugation (6,000 × g at 4°C for 10 min) at 0 and 2 h after the addition of 0.4 μg/mL CFDC and then washed thrice with 1 M sterile precooled phosphate-buffered saline (PBS) (pH = 7.4). All samples were lysed using 8 M urea lysis buffer with intermittent sonication. The protein concentration was quantified using a BCA protein assay kit (Thermo Scientific, USA). The total proteins (200 μg) from each sample were reduced with 50 mM dithiothreitol (DTT) for 1 h at 37°C and then alkylated with 100 mM iodoacetamide (IAA) at room temperature for 30 min in the dark. Subsequently, proteins were digested with trypsin (Huashili, Beijing, China) (protein/trypsin, 30:1, g/g) for 14 h at 37°C. Peptides were dried with a cold-trap speed vacuum concentrator. The dried peptides were resuspended in deionized water containing 0.1% (vol/vol) formic acid. The pooled peptides collected from all samples of equivalent volume were preisolated into 6 fractions as previously described (25, 52).

### Data-independent acquisition mass spectrometry.

DIA and data-dependent acquisition (DDA) MS analysis were used as previously described with some modification (25). Briefly, an iRT-kit (Biognosys, Schlieren, Switzerland) was employed in the peptide sample at a 1:10 ratio to correct the retention time. DDA- and DIA-MS analyses were performed with an Orbitrap Fusion Lumos mass spectrometer (Thermo, USA) equipped with an EASY-nLC 1000 (Thermo, USA). The peptides were separated on Omics high-resolution series monolithic capillary high-pressure liquid chromatography (HPLC) columns (100 μM × 50 cm; Kyoto Monotche) with a column temperature of 50°C. The MS parameters were performed as described previously (25). Raw DDA data sets were used to search against the UniProt E. coli K-12 database (4,356 entries) in the Sequest HT (Proteome Discoverer v2.2) local server and Biognosys Spectronaut software Pulsar (25). The DIA search parameters were also performed as described previously (25).

### Bioinformatics analysis.

The DEPs were analyzed by Cytoscape software (version 3.8.3) with various plug-ins as previously reported (26). The ClueGO v2.5.7 + CluePedia v1.5.8 plug-in was used to discern pathway networks to identify core functional units. Searches were performed against the GO-Biological Process (3,023 terms, 7,587 available unique genes), GO-Molecular Function (2,591 terms, 7,183 available unique genes), and KEGG databases (105 pathways, 1,582 available unique genes) with evidence from all experimental codes (EXP, IDA, IPI, IGI, IEP). Herein, the *P* value was set as two-sided hypergeometric tests and adjusted via Bonferroni correction, and *P *< 0.05 was considered statistically significant. In KEGG pathway analysis, the ProteinRatio = the numbers of the DEPs in one KEGG pathway/the whole numbers of proteins in one KEGG pathway.

### Validation of protein expression by using PRM.

Three unique peptides (unmodified, 0 missing cleavages) per candidate protein were selected for PRM quantification. The chromatographic conditions were the same as those in the DIA experiment. The parameters of Orbitrap Fusion Lumos mass spectrometry were set as follows: MS1 scan range was 350 to 1,550 *m/z*, the resolution was 60,000, automatic gain control (AGC) target was 4e5, and MS2 acquisition used the target ms2 module to monitor the target *m/z* list (see Table S3 in the supplemental material) with a resolution of 30,000, isolation window of 1.6 Da, 5e4 AGC target and maximum injection time of 120 ms, and HCD collision energy of 35. The PRM raw data were loaded into Protein Discoverer 2.2 (Thermo Scientific, MA, USA) to perform peptide identification, and the pdResults file containing peptide spectra was read by Skyline 20.1.0 (53). With a cutoff score of >0.9, 7 amino acid (aa) < peptide length <30 aa, and ion types b, y, and p, Skyline 20.1.0 was used to build the translation list and spectral library, and 3 products with dot *P* values greater than 0.8 were used for peptide quantification and protein quantification.

### Motility assays.

The motility was monitored on semisolid agarose (0.5%) with 0, 0.4, and 0.8 μg/mL CFDC in accordance with our previous work (54, 55). Single bacterial colonies were seeded on agarose plates, and then the plates were cultured for 18 h at 37°C. Finally, the data were quantified by digitally measuring the diameter of the colonies.

### Measurement of reactive oxygen species.

Intracellular ROS were probed using DCFH-DA (2,7-dichlorodihydrofluorescein diacetate) and measured using a BioTek Synergy 2 microplate reader according to the kit's instructions (Beyotime Co., China). Briefly, E. coli BW25113 cells were grown in LB medium to an OD_600_ of ~0.6, treated with or without CFDC, CPM, CAZ, or 20 μM H_2_O_2_ (positive control) for 2 h and then washed three times with 1 M sterile precooled PBS. DCFH-DA was added to each well at a 1:200 ratio and incubated for 30 min at 37°C in the dark. After washing thrice with 1 M sterile precooled PBS, the levels of ROS in bacterial cells were detected at fluorescence excitation/emission wavelengths of 485 ± 20 nm/525 ± 20 nm and then normalized to the corresponding sample protein concentration.

### Measurement of NADH.

The NADH content was measured using an NAD^+^/NADH assay kit with the WST-8 method (Beyotime, Nantong, China) according to the manufacturer’s instructions. Briefly, E. coli BW25113 was grown in LB medium to an OD_600_ of ~0.6, cell pellets were harvested after treatment with 0, 0.4, or 0.8 μg/mL CFDC for 2 h, and then bacterial cells were lysed with 200 μL precooled lysis buffer. The lysed cell suspension was incubated at 60°C for 30 min, and then 20 μL supernatant with 90 μL alcohol dehydrogenase was added to a 96-well plate. The plate was incubated at 37°C for 10 min protected from the light. Finally, 10 μL chromogenic solution was added to the mixture, and the plate was incubated at 37°C for 30 min. A standard curve was generated and measured as the samples. NADH content was measured at 450 nm via a BioTek Synergy 2 microplate reader and then normalized to the sample protein concentration.

### Measurement of H_2_O_2_.

The H_2_O_2_ activity was measured using the Amplex Red hydrogen peroxide/peroxidase assay kit (A22188; Thermo Fisher, USA). Briefly, E. coli BW25113 was grown in LB medium to an OD_600_ of ~0.6, treated with 0, 0.6, or 0.8 μg/mL CFDC, 0.8 μg/mL CFDC +0.2/+0.4/+0.8 mg/mL DFO, or 20 μM H_2_O_2_ (positive control) for 2 h and then washed thrice with 1 M sterile PBS at room temperature. Immediately thereafter, all samples were prepared according to the manufacturer’s instructions. The H_2_O_2_ activity was measured using a BioTek Synergy 2 microplate reader with excitation at 530 ± 25 nm and fluorescence emission monitoring at 590 ± 35 nm and then normalized to the corresponding sample protein concentration.

### Measurement of iron contents.

E. coli BW25113 cells were grown in LB medium to an OD_600_ of ~0.6, and cell pellets were harvested after treatment with 0, 0.4, or 0.8 μg/mL CFDC for 2 h. The iron concentration was measured by using an Iron assay kit (MAK025; Sigma, USA) according to the manufacturer’s protocol. Briefly, bacterial cells were suspended in 1 M sterile PBS and homogenized using multiple freeze and thaw cycles, and 30 μL supernatant sample and 70 μL iron assay buffer were added to a 96-well plate. To measure total iron Fe^2+^ or total iron, 5 μL iron assay buffer or 5 μL iron reducer was added to each well. All samples were incubated at 25°C for 30 min protected from the light. Then, 100 μL iron probe was added to the mixture at 25°C for 60 min and protected from light. Thereafter, the absorbance was detected at 593 nm using a BioTek Synergy 2 microplate reader and then normalized to the corresponding sample protein concentration.

### Statistics.

Data were analyzed using two-tailed, unpaired Student *t* tests or one-way analysis of variance (ANOVA) test with Dunnett's correction and expressed as means ± the standard deviations (SD). Statistical analysis was conducted using Prism 8.0 (GraphPad Software, USA). Results were considered significant at a *P *value of <0.05.

### Data availability.

The raw data and search results have been deposited to the ProteomeXchange Consortium via the PRIDE partner repository with the data set identifier PXD031624.
