# Supplementary material for: Proteomic Investigation of the Antibacterial Mechanism of Cefiderocol against Escherichia coli
Source: Microbiol Spectr. 2022 Aug 18;10(5):e01093-22. doi: 10.1128/spectrum.01093-22 (PMC9603102; doi:10.1128/spectrum.01093-22)
Supplement: Supplemental file 1 — Table S1, Table S2, and Fig. S1. Download spectrum.01093-22-s0001.pdf, PDF file, 0.4 MB [file spectrum.01093-22-s0001.pdf]

SUPPLEMENTAL MATERIAL

Table S1. DEPs identified by proteomics.

Table S2. DEPs involved in catalytic synthesis of NADH, FADH and NADPH, and conversion of NADPH to NADH.

FIG S1. (A) Levels of cell motility with or without CFDC treatment. (B) Levels of Fe^{2+}, Fe^{3+} and total iron ions (Fe^{2+} and Fe^{3+}) in cells with or without CPM/CAZ treatment. Data in this figure was analyzed by using one-way ANOVA test with Dunnett's correction and error bars indicate SD values. $*P < 0.05$, $**P < 0.01$.

Table S1. DEPs identified by proteomics

Acession	Gene	P_value	Fold Change
P0A9G4	cueR	0.0051	0.155
P28630	holA	0.0132	0.178
P00895	trpE	0.0256	0.248
P39358	yjhG	0.0165	0.346
P75937	flgE	0.0001	0.427
P0ABX2	flgC	0.0002	0.458
P0A6X1	hemA	0.0104	0.465
P11868	tdcD	0.0013	0.472
P19319	narZ	0.0459	0.497
P04128	fimA	0.0103	0.513
P77162	ykfB	0.0115	0.524
P0A8S9	flhD	0.0035	0.529
P00634	phoA	0.0479	0.534
P04949	fliC	0.0358	0.538
P0AAE8	cadB	0.0009	0.550
P75938	flgF	0.0005	0.555
A0A385XJ53	insA9	0.0095	0.575
P37348	yecE	0.0062	0.577
P39356	yjhU	0.0037	0.578
P0ABW9	flgB	0.0004	0.584
P39408	yjjV	0.0246	0.593
P39304	ulaD	0.0313	0.623
P0A8L5	ycgN	0.0488	0.626
P0A9W3	ettA	0.0196	0.636
P0A9C0	glpA	0.0252	0.650
P0AFX7	rseA	0.0005	0.660
P18196	minC	0.0034	0.660
P33355	yehS	0.0147	0.670
P11447	argH	0.0405	0.671
P0AEN4	ftsL	0.0416	0.677
P0ABX5	flgG	0.0110	0.678
P0AD70	ampH	0.0095	0.688
P0A725	lpxC	0.0000	0.691
P26608	fliS	0.0182	0.697
P0AAB4	ubiD	0.0115	0.708
P52627	fliZ	0.0341	0.709
P75936	flgD	0.0102	0.713
P77202	dsbG	0.0160	0.714
P0A917	ompX	0.0130	0.714
P0ACB2	hemB	0.0073	0.724
P77375	ydhX	0.0047	0.727
P05706	srlB	0.0018	0.733
P0A6S3	flgI	0.0118	0.734
P29744	flgL	0.0086	0.735
P24178	yffB	0.0020	0.739
P77475	yqaB	0.0200	0.739
P00562	metL	0.0182	0.740
P0A9I8	nirD	0.0459	0.740
P31550	thiB	0.0011	0.745
P0AEW6	gsk	0.0451	0.749
P06996	ompC	0.0029	0.754
P22939	ispA	0.0283	0.763

P0AAL6	ydhY	0.0113	0.764
P0AEW4	cpdA	0.0272	0.768
P0AGE0	ssb	0.0327	0.768
P0ACU7	yjdC	0.0308	0.771
P0A9I3	gcvR	0.0212	0.775
P0A8I8	rlmH	0.0460	0.776
P0AG76	sbcD	0.0212	0.776
P23874	hipA	0.0394	0.777
P46889	ftsK	0.0354	0.784
P00811	ampC	0.0199	0.786
P33644	yfiH	0.0097	0.786
P24228	dppA	0.0266	0.794
P0A9J6	rbsK	0.0064	0.794
P76055	ttcA	0.0187	0.794
P39172	znuA	0.0393	0.795
P00888	aroF	0.0464	0.796
P0AGJ2	trmH	0.0307	0.796
P02943	lamB	0.0143	0.800
P23522	garL	0.0019	0.804
P14175	proV	0.0134	0.808
P66948	bepA	0.0002	0.810
P0ADN2	yifE	0.0195	0.816
P0AF93	ridA	0.0428	0.819
P0A6E9	bioD2	0.0103	0.823
P21338	rna	0.0357	0.823
P68066	grcA	0.0100	0.827
P61175	rplV	0.0453	0.829
P03007	dnaQ	0.0234	0.830
P76149	sad	0.0271	0.833
P77522	sufB	0.0159	1.201
Q46845	yghU	0.0277	1.210
P24251	crl	0.0381	1.211
P0ADE6	kbp	0.0051	1.214
P77335	hlyE	0.0115	1.215
P39176	erfK	0.0388	1.218
P0AC33	fumA	0.0184	1.224
P0ABH9	clpA	0.0049	1.230
P77689	sufD	0.0023	1.230
P05042	fumC	0.0001	1.231
P76329	yedP	0.0353	1.233
P0A6V1	glgC	0.0042	1.233
P50466	aer	0.0087	1.234
P0AGK1	ubiA	0.0314	1.236
P0AD68	ftsI	0.0358	1.238
P0ACS7	rpiR	0.0173	1.243
P07014	sdhB	0.0062	1.247
P0A867	talA	0.0252	1.252
P42620	yqjG	0.0007	1.254
P0AAG8	mglA	0.0184	1.261
P0AAS0	ylaC	0.0231	1.263
P0AFG6	sucB	0.0138	1.265
P31677	otsA	0.0008	1.270
P0AAT9	ybeL	0.0011	1.279
P27306	sthA	0.0230	1.279
P77223	rsxB	0.0167	1.280
P76145	tam	0.0111	1.288

P0AGK4	yhbY	0.0184	1.291
P07003	poxB	0.0152	1.312
P0AFG3	sucA	0.0026	1.318
P26602	ubiC	0.0247	1.340
P0A836	sucC	0.0266	1.351
P0ACD8	hyaB	0.0117	1.352
P0A991	fbaB	0.0047	1.353
P31806	nnr	0.0244	1.359
P69739	hyaA	0.0263	1.362
P52095	ldcC	0.0154	1.364
P0AG84	yghA	0.0008	1.368
P0AC41	sdhA	0.0001	1.371
P21367	ycaC	0.0048	1.374
P25748	galS	0.0396	1.378
P0ABE2	bolA	0.0180	1.387
P0AGE9	sucD	0.0262	1.388
P15877	gcd	0.0411	1.392
P76193	ynhG	0.0075	1.392
Q46857	dkgA	0.0250	1.397
P77674	ydcW	0.0314	1.399
P64624	yheO	0.0016	1.401
P37313	dppF	0.0103	1.406
P76503	fadI	0.0034	1.406
P0ABT2	dps	0.0073	1.413
P39342	yjgR	0.0027	1.417
P64517	yodC	0.0418	1.427
P0ACY3	yeaG	0.0046	1.446
P76402	yegP	0.0237	1.452
P0AFX4	rsd	0.0317	1.470
P0ABH7	gltA	0.0019	1.478
P37902	gltI	0.0421	1.485
P0AE48	ytfP	0.0476	1.493
P65807	ygeY	0.0018	1.505
P0AFH8	osmY	0.0366	1.505
P20605	fic	0.0048	1.555
P0A6J5	dadA	0.0025	1.566
P0A830	dctA	0.0480	1.575
P69451	fadD	0.0202	1.591
Q46799	xdhA	0.0096	1.601
P0AAG0	dppD	0.0392	1.608
P16681	yjdN	0.0065	1.627
P0A756	kefG	0.0221	1.665
P25516	acnA	0.0062	1.669
P69910	gadB	0.0047	1.670
P76108	ydcS	0.0025	1.673
P25553	aldA	0.0046	1.683
P23847	dppA	0.0166	1.689
P39325	ytfQ	0.0059	1.708
P0ADL3	yicN	0.0066	1.738
P10384	fadL	0.0092	1.774
P29012	dadX	0.0247	1.782
P37330	glcB	0.0395	1.793
P24242	ascG	0.0070	1.800
P66899	ygeX	0.0007	1.804
P13445	rpoS	0.0024	1.843
P0ADX5	yhfG	0.0203	1.852

P69908	gadA	0.0023	1.864
P0AFW2	rmf	0.0002	1.871
P25526	gabD	0.0047	1.880
Q47146	fadE	0.0040	1.892
P29013	ycgB	0.0005	1.893
Q46814	xdhD	0.0001	1.907
P0A901	blc	0.0089	1.922
P76097	ydcJ	0.0010	1.926
P0A9G6	aceA	0.0318	1.967
Q46812	ssnA	0.0023	2.031
P63235	gadC	0.0014	2.046
P08997	aceB	0.0122	2.050
P07117	putP	0.0364	2.103
P37685	aldB	0.0009	2.121
P0ACL5	glcC	0.0008	2.162
P77569	mhpR	0.0197	2.191
P0A6J1	cysC	0.0431	2.288
P33595	sgrR	0.0024	2.307
P10907	ugpC	0.0150	2.369
P76142	lsrB	0.0028	2.437
P76143	lsrF	0.0003	2.439
P22256	gabT	0.0001	2.502
P31133	potF	0.0104	2.549
P09546	putA	0.0061	2.724
P37339	lhgD	0.0011	2.834
P09551	argT	0.0103	2.920
P42588	patA	0.0000	3.144
P21177	fadB	0.0002	4.374
P76621	glaH	0.0009	7.460
P27550	acs	0.0003	9.632

Table S2. DEPs involved in catalytic synthesis of NADH, FADH and NADPH, and conversion of NADPH to NADH

Metabolic pathways	Protein	Biochemical Reaction	P_value	Fold Change
Tricarboxylic acid cycle	SucA	2-oxoglutarate + coenzyme A + NAD^+ → succinyl-CoA + CO_2 + NADH	0.0026	1.318
	SucB	2-oxoglutarate + coenzyme A + NAD^+ → succinyl-CoA + CO_2 + NADH	0.0138	1.265
Lysine degradation	YdcW	4-aminobutanal + H_2O + NAD^+ = 4-aminobutanoate + 2 H^+ + NADH	0.0314	1.399
Pyruvate metabolism	AldA	(S)-lactaldehyde + NAD^+ + H_2O → (S)-lactate + NADH + 2 H^+	0.0046	1.683
	PutA	L-glutamate-5-semialdehyde + NAD^+ + H_2O → L-glutamate + NADH + 2 H^+	0.0061	2.724
Tricarboxylic acid cycle	SdhA	FAD + succinate = $FADH_2$ + fumarate	0.0001	1.371
	SdhB	FAD + succinate = $FADH_2$ + fumarate	0.0062	1.247
Fatty acid degradation	FadE	a medium-chain 2,3-saturated fatty acyl-CoA + H^+ + FAD= a medium-chain trans-(2E)-enoyl-CoA + $FADH_2$	0.0040	1.892
	GabD	H_2O + $NADP^+$ + succinate semialdehyde = 2 H^+ + NADPH + succinate	0.0047	1.880
Tricarboxylic acid cycle	SthA	NAD^+ + NADPH = NADH + $NADP^+$	0.0230	1.279

Fig S1

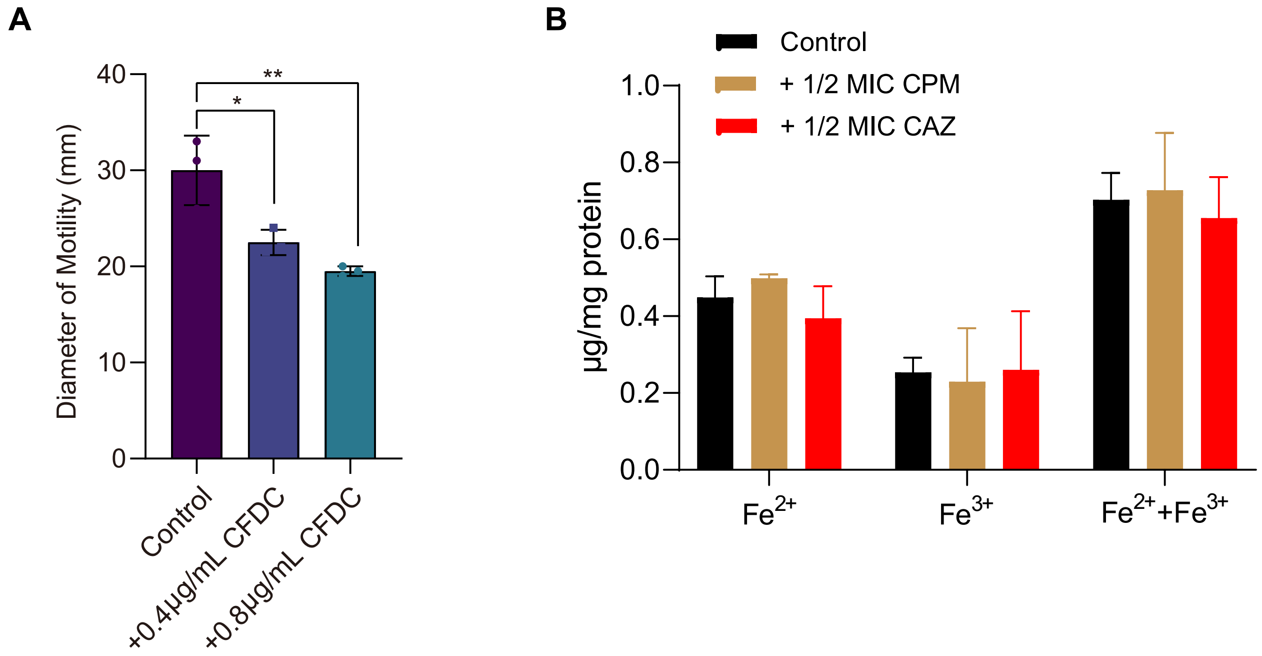
A
B
Diameter of Motility (mm)
40
30
20
10
0
*
**
Control
+0.4μg/mL CFDC
+0.8μg/mL CFDC
μg/mg protein
1.0
0.8
0.6
0.4
0.2
0.0
Control
+ 1/2 MIC CPM
+ 1/2 MIC CAZ
Fe2+
Fe3+
Fe2++Fe3+
